# Analysis of the current status and influencing factors of LEDVT in patients with acute hemorrhagic stroke

**DOI:** 10.1097/MD.0000000000041759

**Published:** 2025-03-21

**Authors:** Xuemei Chen, Zhao Sui, Jing Ting, Minjin Qi, Yijing Yin, Furong He

**Affiliations:** a Department of Neurosurgery, The First Affiliated Hospital of Chengdu Medical College, Chengdu, Sichuan, China.

**Keywords:** acute hemorrhagic stroke, lower extremity venous thrombosis, prognosis, quality of life, risk factors

## Abstract

Lower extremity deep venous thrombosis (LEDVT) is a common complication in patients with acute hemorrhagic stroke, leading to increased risk of pulmonary embolism, disability, and mortality. Despite its importance, LEDVT often goes undetected in clinical practice, and early preventive strategies remain insufficient. This study aimed to explore the incidence of LEDVT in acute hemorrhagic stroke patients, identify key risk factors, and discuss potential preventive measures to reduce its occurrence and improve patient outcomes. A retrospective analysis was conducted on 431 acute hemorrhagic stroke patients admitted to The First Affiliated Hospital of Chengdu Medical College between January 2022 and December 2023. Relevant clinical data, including patient demographics, comorbidities, NIHSS score, and treatment history, were collected. LEDVT was diagnosed using standardized ultrasound criteria. Statistical analyses, including univariate and multivariate logistic regression, were performed using SPSS 17.0 to identify independent risk factors associated with LEDVT. The incidence of LEDVT among the 431 acute hemorrhagic stroke patients was 12.1%, with 52 cases identified. Significant risk factors for LEDVT included advanced age, diabetes, infection, prolonged bed rest, high-dose diuretic use, NIHSS score ≥16, and hyperlipidemia (*P* < .05). Gender, smoking history, and alcohol consumption were not found to be statistically significant. Multivariate logistic regression revealed that advanced age, diabetes, infection, prolonged bed rest, high-dose diuretic use, NIHSS score ≥16, and hyperlipidemia were independent risk factors for LEDVT. LEDVT in acute hemorrhagic stroke patients is influenced by multiple factors, including comorbidities, severity of neurological impairment, and treatment regimens. Dehydration therapy used for managing brain edema and intracranial pressure was also found to be an independent risk factor. Given the significant impact of LEDVT on patient prognosis, early identification of at-risk patients and the implementation of proactive preventive measures—such as pharmacological treatments and physical interventions—are critical in reducing the occurrence of LEDVT, alleviating patient suffering, and improving long-term outcomes. Future studies should focus on refining preventive strategies and exploring more individualized interventions to further reduce the incidence of LEDVT in these patients.

## 1. Introduction

Hemorrhagic stroke is a common and severe cerebrovascular event, with high incidence, disability, mortality, and recurrence rates. It accounts for 10% to 20% of all strokes in Western countries and approximately 20% to 30% in China.^[1,2]^ Despite advances in treatment, hemorrhagic stroke remains a major clinical challenge due to the complexity of management and risk of complications. Current treatments mainly focus on controlling blood pressure with antihypertensive medications such as labetalol or nicardipine, maintaining systolic blood pressure (SBP) below 140 to 160 mm Hg, and using antifibrinolytic agents like tranexamic acid or recombinant activated factor VII to manage bleeding.^[3,4]^ However, these treatments have limitations, including risks of exacerbating coagulation disturbances and creating an imbalance between thrombotic and hemorrhagic events.

A major complication of acute hemorrhagic stroke is lower extremity deep venous thrombosis (LEDVT), where abnormal coagulation in the deep veins of the lower limbs leads to venous obstruction and impaired blood flow. Thrombi formed in the deep veins can dislodge and cause pulmonary embolism, together with deep vein thrombosis (DVT), forming venous thromboembolism, which can significantly impact patient outcomes. The consequences of DVT include pulmonary embolism, post-thrombotic syndrome, and other long-term complications, severely affecting quality of life and potentially leading to mortality.^[5–7]^

Risk factors for DVT are classified into primary and secondary categories. Primary factors include genetic and acquired conditions such as deficiencies in antithrombin, congenital abnormalities in fibrinogen, and mutations in coagulation factors like Factor V Leiden and Factor II 20210A.^[8,9]^ Secondary factors include stroke, prolonged bed rest, advanced age, immobilization, and malignancy.^[10,11]^ Stroke itself is a well-established risk factor for LEDVT due to venous damage, blood stasis, and hypercoagulability, which contribute to thrombus formation. However, despite recognizing stroke as a risk factor, preventing LEDVT in acute hemorrhagic stroke management remains insufficient, particularly in patients receiving treatments like dehydration agents and interventional therapies to restore vessel patency.

Existing approaches to LEDVT prevention often involve the use of pharmacological anticoagulants and physical methods like compression devices. However, these techniques have notable drawbacks. Pharmacological agents can increase the risk of bleeding in stroke patients, particularly in those with active hemorrhage, and physical measures such as early mobilization or pneumatic compression devices may not always be feasible due to patient’s neurological deficits and impaired mobility. These challenges underscore the need for more targeted and effective prevention strategies that consider the unique characteristics and risks of acute hemorrhagic stroke patients.

The motivation behind this study is to address these gaps in the existing literature by exploring the risk factors for LEDVT in acute hemorrhagic stroke patients and evaluating how specific treatments may influence thrombotic events. By conducting a retrospective analysis of 431 cases of acute hemorrhagic stroke admitted to our institution, this study aims to identify key risk factors for LEDVT that could help clinicians better understand which patients are at the highest risk for thromboembolic complications. The goal is to enable more proactive and tailored interventions—whether pharmacological, physical, or procedural—to reduce LEDVT occurrence, improve survival outcomes, and enhance the overall quality of life for these patients. Through this study, we hope to contribute valuable data that can guide clinical practice and ultimately improve the management of acute hemorrhagic stroke and its associated complications.

## 2. Subjects and methods

### 2.1. Study subjects

This study was approved by the Ethics Committee of The First Affiliated Hospital of Chengdu Medical College. We selected 431 patients with acute hemorrhagic stroke admitted to our institution from January 2022 to December 2023. Informed consent was obtained from all participants or their families in accordance with ethical guidelines. Since this is a retrospective study, patient confidentiality and data privacy were strictly maintained. Inclusion criteria were as follows: ① All patients met the diagnostic criteria for cerebral hemorrhage outlined in the “Chinese Guidelines for the Prevention and Treatment of Cerebrovascular Diseases”^[12]^; ② Confirmed spontaneous intracerebral hemorrhage by head CT or MRI upon admission; ③ Onset of symptoms within 48 hours before admission; ④ First-ever occurrence of stroke; ⑤ Age ≥18 years old; ⑥ Hospital stay of ≥7 days. Exclusion criteria were: ① Patients presenting with profound coma upon admission (GCS 3–5)^[13]^; ② Patients with poor prognosis due to large hematoma; ③ Patients scheduled for early surgical hematoma evacuation; ④ Patients with severe conditions affecting prognosis such as significant heart, liver, lung, kidney diseases, or traumatic brain hemorrhage.

### 2.2. Methods

We employed a retrospective survey method to collect relevant patient information including: age (categorized as <60 years and ≥60 years), gender, smoking history, alcohol consumption history, length of hospital stay, presence of comorbid tumors, pregnancy status, presence of hypertension, diabetes mellitus, hyperlipidemia, presence of infections, NIHSS score (categorized as < 16 and ≥ 16 according to the National Institutes of Health Stroke Scale^[14,15]^), frequency of diuretic use (categorized as <4 times/day and ≥4 times/day), duration of bed rest (categorized as <14 days and ≥14 days), whether patients underwent interventional therapy, location of lower limb venous thrombosis, and use of medications such as aspirin and clopidogrel, as detailed in Table 1.

**Table 1:** Basic clinical data of patients.

Clinical data		Total cases (431)		LEDVT cases (52)	
Gender	Male	234	54.3%	29	6.7%
	Female	197	45.7%	23	5.3%
Age	60 years old	142	32.9%	9	2.1%
	≥60 years old	289	67.1%	43	10.0%
Smoking history	With	225	52.2%	28	6.5%
	Without	206	47.8%	24	5.6%
Drinking history	With	257	59.6%	32	7.4%
	Without	174	40.4%	20	4.6%
Hypertension	With	293	68.0%	44	10.2%
	Without	138	32.0%	8	1.9%
Diabetes	With	106	24.6%	22	5.1%
	Without	325	75.4%	30	7.0%
Hyperlipidemia	With	153	35.5%	27	6.3%
	Without	278	64.5%	25	5.8%
Tumour	With	5	1.2%	3	0.7%
	Without	426	98.8%	49	11.4%
Gestation	With	0	0.0%	0	0.0%
	Without	431	100.0%	52	12.1%
Infect	With	305	70.8%	45	10.4%
	Without	126	29.2%	7	1.6%
NIHSS score	<16	248	57.5%	16	3.7%
	≥16	183	42.5%	36	8.4%
Bedtime	<14 d	179	41.5%	14	3.2%
	≥14 d	252	58.5%	38	8.8%
Interventional therapy	With	108	25.1%	33	7.7%
	Without	323	74.9%	19	4.4%
Taking aspirin	With	132	30.6%	12	2.8%
	Without	299	69.4%	40	9.3%
Taking clopidogrel	With	288	66.8%	26	6.0%
	Without	143	33.2%	26	6.0%
Dehydrating agent	<4 times/d	304	70.5%	18	4.2%
	≥4 times/d	127	29.5%	34	7.9%

LEDVT = lower extremity deep venous thrombosis.

In this study, the diagnosis of LEDVT was based on standardized ultrasound criteria.^[16]^ The criteria included the presence of variable echogenicity within the lumen of the deep veins, inability to fully compress the veins or partial compression during the examination, absence of Doppler blood flow or identification of filling defects, and widening of the internal diameter of the veins. To ensure the accuracy and consistency of the diagnostic process, high-resolution portable ultrasound machines equipped with both B-mode and Doppler capabilities were used. All examinations were conducted by trained radiologists experienced in vascular imaging, following a standardized protocol to minimize inter-operator variability. The ultrasound examinations were performed within 72 hours of stroke onset, as this time frame is critical for detecting early thrombus formation. The patients were positioned in a supine position with the leg slightly elevated to enhance venous return, and both femoral, popliteal, and tibial veins were assessed bilaterally. By following this standardized approach, we aimed to reduce diagnostic variability and enhance the reliability of ultrasound in detecting LEDVT.

Clinical prognosis assessment: LEDVT patients were classified based on glasgow outcome scale scores at discharge, with glasgow outcome scale scores of 4 to 5 categorized as the favorable prognosis group and scores of 1 to 3 as the poor prognosis group.^[17]^ The study included 32 cases in the favorable prognosis group and 20 cases in the poor prognosis group. Mean SBP and diastolic blood pressure values were recorded during daytime and nighttime at 7 days post-discharge.

#### 2.2.1. Statistical methods

The collected clinical data were analyzed using SPSS 23.0 statistical software. Continuous variables were expressed as mean ± standard deviation $\left(\bar{x}\pm S\right)$ and analyzed using the *t* test. Categorical variables were presented as frequencies and percentages (%) and analyzed using the chi-square test. Independent risk factors analysis for LEDVT was conducted using multiple logistic regression analysis. All results were considered statistically significant at *P* < .05.

## 3. Results

### 3.1. Incidence of LEDVT in patients with acute hemorrhagic stroke

Among the 431 patients included in this study with acute hemorrhagic stroke, 52 cases of LEDVT were detected through lower limb vascular ultrasound, yielding an incidence rate of 12.1%. Among these cases, 29 (55.8%) were male and 23 (44.2%) were female (Table 1).

### 3.2. Comparison of mean blood pressure values between day 7 and day 1 in different prognostic groups of patients with acute hemorrhagic stroke

Of the 52 patients with newly diagnosed acute hemorrhagic stroke, 30 (57.69%) had initial SBP ≥ 140 mm Hg and diastolic blood pressure ≥ 90 mm Hg. There was a significant decrease in mean 24-hour blood pressure values from day 1 to day 7 in both groups (*P* < .001; see Table 2).

**Table 2:** Comparison of mean 24-hour blood pressure on day 7 and day 1 (*x* ± *s*).

Time	SBP (mm Hg)		DBP (mm Hg)	
	Good prognosis group	Poor prognosis group	Good prognosis group	Poor prognosis group
1st day	134.39 ± 21.52	149 ± 15.27	82.22 ± 12.81	90.44 ± 10.97
On the 7th day	127 ± 17.85	136.85 ± 12.37	78.57 ± 10.84	84.39 ± 9.92
*P*-value	<.001	<.001	<.001	<.001

DBP = diastolic blood pressure, SBP = systolic blood pressure.

### 3.3. Single factor analysis of risk factors in patients with acute hemorrhagic stroke

Fifteen factors including age, gender, smoking history, alcohol consumption history, presence of tumors, hypertension status, diabetes status, hyperlipidemia status, presence of infection, NIHSS score, frequency of diuretic use, duration of bed rest, intervention treatment, aspirin use, and clopidogrel use were analyzed individually. The study results indicated statistically significant differences (*P* < .05) in the incidence of LEDVT across groups stratified by age, presence of tumors, hypertension status, diabetes status, hyperlipidemia status, presence of infection, NIHSS score, frequency of diuretic use, duration of bed rest, intervention treatment, aspirin use, and clopidogrel use, as shown in Table 3. However, there were no statistically significant differences (*P* > .05) in LEDVT incidence between groups stratified by gender, smoking history, and alcohol consumption history.

**Table 3:** Univariate analysis of LEDVT in acute ischemic stroke patients.

Influencing factors		Total cases (n = 431)	LEDVT cases (n = 52)	Percentage (%)	*x*^2^ value	*P*-value
Gender	Male	234	29	12.4%	1.274	.283
	Female	197	23	11.7%		
Age	<60 years old	142	9	6.3%	17.563	.000
	≥60 years old	289	43	14.9%		
Smoking history	Have	225	28	12.4%	0.918	.767
	Nothing	206	24	11.7%		
Drinking history	Have	257	32	12.5%	0.319	.655
	Nothing	174	20	11.5%		
Hypertension	Have	293	44	15.0%	3.842	.034
	Nothing	138	8	5.8%		
Diabetes	Have	106	22	20.8%	16.408	.001
	Nothing	325	30	9.2%		
Hyperlipidemia	Have	153	27	17.6%	4.673	.013
	Nothing	278	25	9.0%		
Tumor	Have	5	3	60.0%	9.794	.000
	Nothing	426	49	11.5%		
Infect	Have	305	45	14.8%	10.941	.003
	Nothing	126	7	5.6%		
NIHSS score	<16	248	16	6.5%	6.245	.015
	≥16	183	36	19.7%		
Bedtime	<14 d	179	14	7.8%	9.174	.003
	≥14 d	252	38	15.1%		
Interventional therapy	Have	108	33	30.6%	5.652	.001
	Nothing	323	19	5.9%		
Aspirin	Have	132	12	9.1%	12.362	.000
	Nothing	299	40	13.4%		
Clopidogrel	Have	288	26	9.0%	9.684	.008
	Nothing	143	26	18.2%		
Dehydrating agent	<4 times/d	304	18	5.9%	20.413	.000
	≥4 times/d	127	34	26.8%		

LEDVT = lower extremity deep venous thrombosis.

### 3.4. Multivariable logistic regression analysis of risk factors in patients with acute hemorrhagic stroke

The 12 factors identified from single factor analysis were subjected to multivariable logistic regression analysis. The results indicated that advanced age, diabetes mellitus comorbidity, presence of infection, prolonged bed rest, high-dose diuretic use, NIHSS score ≥16 points, and hyperlipidemia were independent risk factors associated with LEDVT in patients with acute hemorrhagic stroke (*P* < .05), as shown in Table 4.

**Table 4:** Multivariate logistic analysis of LEDVT in acute ischemic stroke patients.

Influencing factors	Regression coefficient	Standard error	Wald value	*P*-value	OR value	95% CI
Age	1.927	0.315	10.834	0.001	3.630	1.498–7.359
Bedtime	1.107	0.241	5.613	0.011	2.264	1.138–4.413
Diabetes	0.983	0.509	3.871	0.027	5.857	2.734–10.574
Infect	0.869	0.417	6.879	0.007	1.851	1.169–7.618
Dehydrating agent	1.211	0.231	6.736	0.019	2.171	0.754–13.452
NIHSS score	1.676	0.799	4.635	0.001	9.868	3.409–19.336
Hyperlipidemia	0.732	0.473	7.69	0.015	3.595	2.076–8.53

LEDVT = lower extremity deep venous thrombosis.

## 4. Discussion

LEDVT is one of the complications associated with acute hemorrhagic stroke, with stroke itself being a risk factor for LEDVT. The detachment of thrombi formed in the deep veins of the lower extremities can lead to pulmonary embolism, collectively known as venous thromboembolism.^[18]^ Therefore, vigilance is crucial to detect LEDVT in patients with acute hemorrhagic stroke. Studies have shown that approximately 300,000 patients die from venous thromboembolism annually in the United States, and about 500,000 in Europe.^[19,20]^ Golomb et al reported that the risk of deep vein thrombosis exceeds 50% in hospitalized patients, yet fewer than half of these high-risk patients ultimately receive preventive treatment, highlighting inadequate and untimely preventive measures.^[21]^ For patients with acute hemorrhagic stroke, the incidence of symptomatic DVT is low, around 2%. Moreover, some patients may develop LEDVT early without manifesting symptoms. Therefore, identifying risk factors for LEDVT in patients with acute hemorrhagic stroke is of paramount importance to implement effective preventive measures promptly. The classical Virchow triad posits that vascular endothelial injury, venous stasis, and hypercoagulability are the 3 major factors contributing to DVT, a theory widely accepted by scholars. This study, through retrospective analysis, aimed to identify risk factors for LEDVT in patients with acute hemorrhagic stroke. The results revealed that advanced age, diabetes mellitus comorbidity, presence of infection, prolonged bed rest, high-dose diuretic use, high NIHSS score, and hyperlipidemia were independent risk factors for LEDVT in these patients. When these risk factors coexist in patients, heightened awareness is warranted to consider early and appropriate preventive measures to reduce the incidence of LEDVT.

In this study, advanced age is identified as a risk factor for LEDVT in patients with acute hemorrhagic stroke. The incidence of LEDVT in acute hemorrhagic stroke patients aged ≥60 years was 14.9%, whereas it was 6.3% in those aged <60 years. Previous studies have demonstrated an increasing incidence of deep venous thrombosis (DVT) with advancing age, particularly rising rapidly after the age of 45, with age over 60 being an independent risk factor for DVT,^[22]^ consistent with the findings of this study. For elderly patients, aging contributes to vascular wall thickening, reduced elastin content, diminished vascular elasticity, and impaired venous return. Additionally, age-related increases in platelet adhesion lead to heightened blood viscosity and reduced blood flow velocity. Aging also decreases calf muscle tone and impairs the muscle pump function, further slowing blood circulation. Karasu et al suggest that elderly patients are more prone to developing deep venous thrombi not only due to these factors but also because aging thickens venous valves and compromises venous function. Moreover, advancing age decreases anticoagulant enzyme levels, reduces fibrinolytic activity, increases clotting factors and fibrinogen, and disrupts normal coagulation function,^[23]^ collectively promoting the formation of lower extremity deep venous thrombosis in elderly patients. Further corroborating this, Geerts et al found that the average age of patients with lower extremity deep venous thrombosis was 58.4 years.^[24]^

In this study, a high NIHSS score is also identified as an independent risk factor for LEDVT in patients with acute hemorrhagic stroke. According to the results of this study, based on the NIHSS scoring criteria, we understand that a higher NIHSS score indicates more severe neurological impairment in patients. Such patients often exhibit more severe paralysis, reduced language expression ability, and diminished self-care capabilities. Greater paralysis leads to weaker lower limb muscle strength and diminished muscle pump function, resulting in slowed blood flow, which promotes thrombus formation. Muir et al also suggest that acute hemorrhagic stroke patients experience significant limb paralysis and notably reduced lower limb muscle strength due to neurological damage, resulting in diminished compressive force on the deep veins of the lower limbs, slow blood flow, and subsequent formation of lower extremity deep venous thrombosis.^[25]^ Arya et al found that patients with paralysis and prolonged bed rest, especially when combined with other risk factors for LEDVT, experience a significantly increased incidence of lower extremity deep venous thrombosis.^[26]^

The study results indicate that diabetes mellitus is an independent risk factor for LEDVT in patients with acute hemorrhagic stroke. Diabetic patients are prone to endothelial cell damage due to prolonged hyperglycemia and abnormal lipid metabolism. When endothelial cells are damaged, they release various vasoactive substances that promote venous thrombus formation. Increased blood glucose concentrations enhance platelet and red blood cell adhesion, leading to a hypercoagulable state. Moreover, diabetes often accompanies vascular changes, including increased vascular fragility, blood viscosity, and slowed blood flow, which facilitate venous thrombosis.^[27]^ Furthermore, diabetes mellitus impairs endothelial nitric oxide synthase activity, reducing nitric oxide secretion and thereby hindering prostaglandin synthesis. This results in decreased vasodilatory substances, endothelial dysfunction, and promotes venous thrombus formation.^[28]^ Diabetes mellitus not only serves as a risk factor for LEDVT in patients with acute hemorrhagic stroke but also predisposes individuals to acute hemorrhagic stroke itself. Therefore, adequate attention should be paid to diabetic patients, focusing on glycemic control and early consideration of LEDVT prevention. In this study, infection is identified as an independent risk factor for LEDVT in patients with acute hemorrhagic stroke. Khorana et al suggest that concomitant infection is a contributing factor to LEDVT.^[29]^ They propose that the release of inflammatory substances during infection activates cytokines, altering blood flow dynamics and promoting a hypercoagulable state conducive to deep vein thrombosis formation. Fever accompanying infection leads to fluid loss, increasing blood viscosity and the risk of thrombosis. Previous research has highlighted multiple interactions between the inflammatory response and the coagulation system.^[30]^ Infection elevates C-reactive protein, enhancing tissue factor expression on leukocytes and promoting platelet reactivity and generation.^[31]^ Esmon et al posit that infection-induced inflammation activates complement, apoptosis, and necrosis, crucial processes in clotting reactions, thereby promoting thrombus formation and potentially damaging venous endothelium.^[32]^

Dehydration therapy is also identified as an independent risk factor for LEDVT in patients with acute hemorrhagic stroke in this study. The incidence of LEDVT was 5.9% in patients receiving dehydration therapy <4 times daily, whereas it was 26.8% in those receiving 4 or more times daily. Dehydration therapy in acute hemorrhagic stroke patients is primarily used to prevent or reduce cerebral edema and intracranial pressure. Hamilton et al demonstrated that hyperosmolar mannitol induces dose-dependent apoptosis in endothelial cells and activates pathways involving clotting and inflammatory cells, contributing to thrombus formation.^[33]^ Therefore, in patients receiving high-dose dehydration therapy, maintaining fluid balance and minimizing blood concentration are crucial to reducing LEDVT incidence. Monitoring the deep vein status of these patients is essential.

Hyperlipidemia is another independent risk factor for LEDVT in patients with acute hemorrhagic stroke in this study. Hur et al found that in patients with hyperlipidemia, fat deposits in vessel walls narrow the vessel lumen, increase blood viscosity, and slow blood flow, ultimately leading to deep vein thrombosis.^[34]^ Ay et al suggested that abnormal lipid metabolism in hyperlipidemia inhibits the fibrinolytic system, increases coagulation factors such as thrombin, factor XII, and factor VII, thereby promoting thrombus formation.^[35]^

This study has several limitations. First, the retrospective design of the study introduces potential bias, including selection bias and information bias. Although retrospective studies provide valuable insights, the lack of randomized control groups and the inability to control for all confounding variables can limit the robustness of the findings. Additionally, the method of collecting the highest NIHSS score may not be optimal, as some patients experience significant early fluctuations in NIHSS scores, which could affect the accuracy of the data. Furthermore, there were a limited number of cases with concomitant tumors in this study, which differs from previous research suggesting tumors as high-risk factors for LEDVT.^[36,37]^ The lack of detailed information regarding the dehydration therapy, including its duration and total amount administered, is another limitation. To address these limitations and minimize bias, future research should consider prospective designs or randomized controlled trials, which would provide stronger evidence and help validate the results. Additionally, a more detailed collection of variables such as NIHSS scores over time and the specific management of dehydration therapy could further enhance the robustness of future studies.

## Author contributions

**Conceptualization:** Xuemei Chen, Zhao Sui, Jing Ting, Minjin Qi, Furong He.

**Data curation:** Jing Ting, Yijing Yin, Furong He.

**Formal analysis:** Xuemei Chen, Jing Ting, Furong He.

**Funding acquisition:** Zhao Sui, Minjin Qi, Furong He.

**Investigation:** Xuemei Chen, Zhao Sui, Jing Ting, Minjin Qi.

**Methodology:** Xuemei Chen, Zhao Sui, Jing Ting, Minjin Qi.

**Software:** Furong He.

**Supervision:** Xuemei Chen, Yijing Yin, Furong He.

**Validation:** Xuemei Chen, Zhao Sui, Yijing Yin.

**Visualization:** Furong He.

**Writing—original draft:** Xuemei Chen, Furong He.

**Writing—review & editing:** Xuemei Chen, Furong He.
